# Relationship Between Socioeconomic Status and Organized Sports Among Primary School Children: A Gender-Based Analysis of Sports Participation

**DOI:** 10.3390/sports13060165

**Published:** 2025-05-28

**Authors:** Chiaki Tanaka, Eun-Young Lee, Shigeho Tanaka

**Affiliations:** 1 Department of Human Nutrition, Tokyo Kasei Gakuin University, 22 Sanbancho, Chiyoda-ku, Tokyo 102-8341, Japan; 2 National Institute of Health and Nutrition, National Institutes of Biomedical Innovation, Health and Nutrition, Settsu, Osaka 566-0002, Japan; tanaka.shigeho@eiyo.ac.jp; 3 Institute of Nutrition Sciences, Kagawa Nutrition University, 3-9-21 Chiyoda, Sakado, Saitama 350-0288, Japan; 4 School of Kinesiology and Health Studies, Queen’s University, Kingston, ON K7L3N6, Canada; eunyoung.lee@queensu.ca; 5 Faculty of Nutrition, Kagawa Nutrition University, 3-9-21 Chiyoda, Sakado, Saitama 350-0288, Japan

**Keywords:** exercise, educational status, children, gender, sport type

## Abstract

Sports participation according to socioeconomic status (SES) was related to children in high-income Western countries. This study aimed to examine whether family or neighborhood-level SES is associated with current and continued organized sports participation, including the types of sports, among Japanese primary school children from preschool onward. The participants consisted of 269 girls, 255 boys, and their parents. Data on the type of sports participation at the current school or preschool, parental employment, and education were collected by questionnaire. Neighborhood-level SES was evaluated by the average annual income within 4 km of each school. The odds of sports participation was higher among children with mothers identifying as housewives or those with mothers employed part-time. Among girls, the odds of continued sports participation were lower if their mothers were junior high school or high school graduates or junior college/vocational school graduates. The odds of sports type like swimming were higher for children whose mothers had part-time jobs. Lower average community income was associated with lower participation in football and higher participation in baseball. These findings suggest that mothers’ employment and academic background are important correlates of sports participation for children, with variations observed by sport type and gender.

## 1. Introduction

Engaging in regular physical activity, including exercise and sports, has been shown to have a positive impact on physical fitness, cardiometabolic health, bone health, cognitive ability (e.g., academic performance), mental health, and social benefits (e.g., improved self-esteem) among children [1]. One of the goals of Healthy Japan 21 (third phase), launched in 2024, is to “Reduce the number of children who do not exercise or play sports habitually” by taking a life course approach [2]. However, a recent national survey conducted by the Sports Agency showed that the proportion of Japanese primary school children who belonged to a sports club was only about 40% for girls and 60% for boys [3].

According to the World Health Organization, health and illness follow a social gradient, with, for example, a lower socioeconomic status (SES) leading to poorer health [4]. Barriers and inequities in opportunities for physical activity may reflect disparities in SES [5]. A recent systematic review and meta-analyses investigating socioeconomic disparities in physical activity and sport participation in high-income countries reported that children and adolescents living in higher SES households were more likely to participate in sports [6]. Furthermore, socioeconomic differences in participation were greater in sports compared to total physical activity and in children compared to adolescents [6]. SES has been described as aspects of the intrapersonal (e.g., education level, employment status, and income of the individual), interpersonal (e.g., education level, employment status, and income of parents or caregivers), and environmental (e.g., profiles of education level, employment status and income for neighborhoods as a whole) [7]. However, to our knowledge, only Yamakita and associates [8] reported the relationship between exercise duration time and parents’ educational background among primary and junior high school students in the Japanese context. However, as mentioned above, SES encompasses not only parents’ educational background but also occupational prestige and income for neighborhoods.

The Sports Agency has prioritized “strengthening efforts to form exercise habits in early childhood” to promote sustained sports participation throughout childhood and beyond [9]. However, although previous studies have examined the relationship between SES and either exercise duration or daily physical activity cross-sectionally, the long-term impact of early sports engagement in relation to SES remains unexplored.

According to the most recent national survey by the Sasakawa Sports Foundation in 2021, the participation rate for preschool children was approximately 50% in 2013 and has remained unchanged since [10]. Swimming had the highest participation rate (20–30%) since 2010, followed by football at around 10%. It has been reported that among Japanese adults, the relationship between SES and sports participation when classifying sports into different types, has been mixed [11]. However, no studies examined such relationships among children. Identifying differential associations between SES and varying types of sports can help better understand the factors influencing participation, ultimately enabling the development of targeted strategies to remove barriers and promote broader access to sports.

The first objective of this research was to examine whether children’s current participation in organized sports or their continued involvement since preschool was associated with household SES factors, including mothers’ and fathers’ educational background and employment status, as well as neighborhood SES. The second objective was to examine the relationship between SES and specific sports participation to identify different associations with SES by different types of sports. We hypothesized that organized sports participation or their continued involvement since preschool would be associated with family or neighborhood-level SES. We further hypothesized that these associations would be different associations with different types of sports.

## 2. Materials and Methods

### 2.1. Participants

In seven public elementary schools in the 23 wards of Tokyo and seven public elementary schools in Kyoto City, 546 children (280 girls and 266 boys) and their mothers (*n* = 440) or fathers (*n* = 266) who agreed to participate in the survey themselves were surveyed. This study was conducted between June 2012 and January 2015. This study was approved by the Research Ethics Committee of J. F. Oberlin University (approval numbers: 10007, 12023). The purpose of the survey, benefits, disadvantages, risks, and publication of data were explained to the participants and their guardians, and written consent was obtained. Assuming an alpha level of 0.05, a power of 0.80, an odds ratio of 2.5, and a sports participation rate of 0.6, the required sample size in order to examine differences in sports participation rate by parental educational background or employment status was calculated to be 215 for each gender using G*Power version 3.1.9.7.

**Figure 1:**
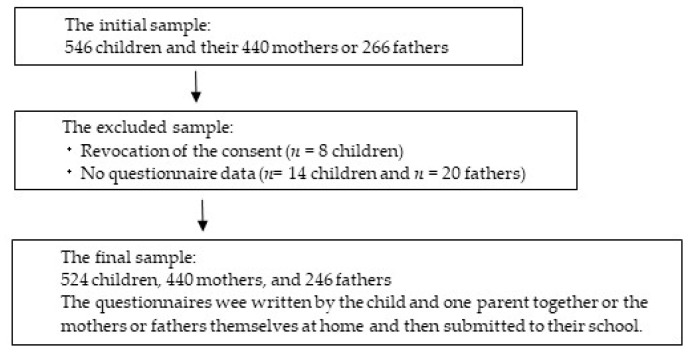
Flow of participants.

Figure 1 shows a flowchart describing the recruitment process and participants’ flow. Of those recruited, 8 children declined to participate in this study, and 14 children and 20 fathers were excluded due to a lack of questionnaire data. Therefore, 524 children, 440 mothers, and 246 fathers were included in this analysis.

### 2.2. Questionnaire

The questionnaire for the children was written by the child and one parent/guardian together at home. The questionnaires for the present study were developed with reference to the standard questionnaire used in Japanese public health surveillance, as the National Survey of Sports Lifestyle conducted by the Sasakawa Sports Foundation [10]. This questionnaire included (1) date of birth, (2) whether the child was currently participating in an extracurricular activity (“Does your child currently participate in an extracurricular activity (e.g., sports, piano, tutoring)?”), and if yes, the contents (up to three types), frequency of participation, and starting age as the current and continued organized sports participation variables; (3) the working status of the mother and father as family SES variables. Children’s demographics (male, female) were collected at each school. The questionnaire for the mothers or fathers who agreed to participate in this study included (1) attributes (father and mother) and (2) their years of education as a family SES variable. The questionnaires were distributed to the children at school in envelopes, which were then submitted to each school eight days after distribution in a sealed envelope. The researcher visited each school to collect the questionnaires.

### 2.3. Statistical Analysis

Sports participants as a dependent variable were defined as those who listed a sport type from the list of lessons in which they regularly participated at least once a week [10].

The relationship between SES and children’s current sports participation was assessed using logistic regression analysis. Sport participation was treated as a binary variable, with the presence of sports participation set to “0” and the absence of sports participation set to “1”. For retrospective examination, sports participants were treated as a dichotomous variable, with the case of continuous participation in one or more sports from early childhood set to “0” and the case of no continuous participation set to “1”.

The results are presented as odds ratios (ORs) and 95% confidence intervals (95% CI). The independent variables used were the parents’ educational background, employment status, and economic status in the neighborhood where they lived [6]. Parental education background was divided into three groups: junior high school or high school graduate equivalent as a low socioeconomic level, junior college or technical college graduate equivalent as a medium socioeconomic level, and university or higher graduate equivalent as a high socioeconomic level, respectively. Mothers’ employment status was classified into four groups: unemployed, including housewives, as a high socioeconomic level, part-time employed as a low socioeconomic level, self-employed as a medium socioeconomic level, and full-time as a high socioeconomic level. Fathers’ employment status was classified into two groups: self-employed as a low socioeconomic level and full-time as a high socioeconomic level, except for one father who reported having a part-time job. The economic situation in the vicinity of the residential area was indicated in the Order for Enforcement of the Act on National Treasury’s Sharing of Expenses for Facilities of Compulsory Education Schools as being within approximately a 4 km radius of the school for elementary schools. Therefore, the average annual household income of residents within a 4 km radius of each target primary school was calculated based on the 2015 Census and the 2013 Housing and Land Survey by the Statistics Bureau of the Ministry of Internal Affairs and Communications, which were conducted by Zenrin Geoinelligence, Inc, Tokyo, Japan. The respondents were further divided into three groups (low, medium, or high socioeconomic levels) according to their average annual income. For the independent variables, full-time for the employment status, the equivalent of a university degree or higher for education, and the group with the highest average income in the region were the reference group, respectively, and employment status and education were simultaneously entered as independent variables. Confounding factors adjusted for the analyses were grade, gender in the analysis for all children, and mother’s age in the analysis of mothers, or father’s age for fathers instead of gender. An interaction term between the independent variables was also included in this model; however, if no significant association was found, the interaction term was excluded, and the OR and 95% CI were calculated. Higher ORs indicate greater sports participation.

Statistical processing was carried out using SPSS package 28.0 J for Windows (IBM Japan Corp., Tokyo, Japan). All statistical significance levels were set at less than 5% two-sided.

## 3. Results

Table 1 shows the characteristics of the participants, children’s sports participation, and participation by sport type. There were no significant differences in sports participation rates by gender or school grade. The rate of continuous participation since preschool children was 70.2% (girls: 77.1%; boys: 64.0%). The highest participation rate was observed for swimming (37.4%).

As the interaction terms between the independent variables were not significant, the results of the analysis are shown without the interaction terms. Table 2 shows the results of the relationship between SES and current sports participation by variables pertaining to mothers and fathers, respectively, for all participants regardless of child gender. The odds of sports participation were higher with mothers reported as housewives (OR: 2.91) or employed part-time (OR: 2.90). By gender, the relationship was only statistically significant among boys only (mothers identified as housewives: OR: 5.69; mothers employed part-time: OR: 6.02). None of the relationships with SES in the vicinity of the area of residence were statistically significant.

The relationship between SES and continued participation in sport among preschool children stratified by gender is shown in Supplementary Material Table S1. The results showed that none of the relationships were significant for all participants. For girls and boys separately, the relationship was statistically significant with mothers with a junior high school or high school degree (OR: 0.25) or junior college degree (OR: 0.27) only for girls. The relationship with the SES variables pertaining to fathers could not be examined among girls due to the insufficient number of participants. No statistically significant relationship was observed between neighborhood-level SES and sports participation.

The relationship between SES and sports participation was examined by each sport type (Table 3). Analysis was conducted for all participants but only for sports with a sufficient number of subjects to allow for logistic regression analysis. The odds of participation in swimming were higher among children with mothers identified as housewives (OR: 2.43) or employed part-time (OR: 2.36) and in football among children with mothers employed part-time (OR: 5.19). In terms of parental education, the odds of participation in swimming among children were lower with mothers reporting a junior high school or high school education (OR: 0.49), and ballet when mothers reported a junior college or vocational school education (OR: 0.07). On the other hand, the odds of participation in gymnastics were higher when fathers had a junior high school or high school education (OR: 5.16). In terms of the relationship between neighborhood-level SES and sports participation by type, the lowest neighborhood-level SES group (OR: 0.39) showed a lower participation rate in football than the group with the highest average household income in the area, and the middle groups had higher participation rates in baseball (OR: 3.02) and gymnastics (OR: 2.96) than the groups with the highest average household income in the area.

## 4. Discussion

The objective of the present study was to examine whether family or neighborhood-level SES is associated with current and continued organized sports participation, including the types of sports, among Japanese primary school children from preschool onward. As a result, the odds of sports participation were higher among children with mothers identifying as housewives or those with mothers employed part-time. Among girls, the odds of continued sports participation were lower if their mothers were junior high school or high school graduates or junior college/vocational school graduates. The odds of sport type such as swimming were higher for children whose mothers had part-time jobs. Lower average community income was associated with lower participation in football and higher in baseball.

With regard to sports participation in childhood, the participation rate for primary school children was about 70% in the nationwide surveys conducted by the Sasakawa Sports Foundation [10]. The participation rate in the present study was comparable to that of a national survey of the Sasakawa Sports Foundation [10]. To the best of our knowledge, no previous studies have reported the participation rates of continuous sports involvement from preschool age in Japan. Moreover, swimming had the highest participation rate of approximately 20–30% for approximately 10 years since 2010 in the Sasakawa Sports Foundation [10]. This was followed by football at around 10%, while the nationwide surveys conducted by the Benesse Institute of Education reported that the highest participation rate was observed for swimming (33.6%), followed by football (8.7%) [12]. The highest participation rate in the present study was observed for swimming, followed by football and gymnastics in our sample, similar to the above surveys [10,12]. As swimming is an important life-saving motor skill, many families may enroll their children in swimming lessons to help them develop essential skills [13].

In our sample, children with mothers identified as housewives or employed part-time engaged in sports more. A similar relationship was found among boys only. However, the father’s employment status or parents’ educational background was not associated with sports participation among children. Yamakita and associates [8] reported that elementary school girls of parents with 13 or more years of education were more likely to exercise less than 7 h per week than elementary school girls of parents with 12 or fewer years of education (OR: 1.30, 95% CI: 1.00 −1.69, *p* = 0.0498) in Japan. However, no significant influences of parents’ educational levels were found for boys, while previous studies in other countries studies by Owen and associates [6] have reported that overall, children and adolescents living in higher socioeconomic status households were more likely to participate in sports (OR: 1.87, 95% CIs 1.38–2.36). Although information on individual income could not be obtained in this study, it is plausible that having a mother with a full-time job or identified as a housewife is related to higher household income. If so, boys with part-time mothers with lower incomes would be more likely to participate in sports, which differs from previous studies. On the other hand, according to a previous study, the higher the annual household income or higher parents’ educational background, the higher the cost of out-of-school educational activities per month tended to be in Japan [12]. In addition, it was reported that boys engage more in sports activities while girls tend to engage more in artistic activities. Furthermore, housewives or mothers with part-time jobs may have more time to take their children to and from sports facilities, which may have resulted in a higher rate of participation in sports by their children. These results may indicate that mothers’ occupations may have a greater influence on boys’ sports participation compared to girls.

In the present study, no relationship was found between the average annual income of the neighborhood and sports participation. In the present study, only the neighborhood-level income was used; therefore, it may not have adequately reflected the socioeconomic composition of the neighborhood of residence.

The relationship between SES and continued participation in sports among preschool children was examined. For girls only, continuous sports participation was associated with maternal education. The breakdown of participation rates for each sport type among girls showed that swimming (33.8%) was the most popular one, followed by ballet and dance (19.7% combined). The combined participation rate for ballet and dance among boys was only 2.4%. Specifically, participation in artistic sports can be inherited from mothers to daughters, as indicated in Bourdieu’s theory of cultural reproduction [14]. In addition, Kwon and associates [15] reported in a US cohort study that the role of fathers may be important in encouraging children from low-income families to continue participating in sports from the ages of 5–19 years. Due to the limited number of fathers included in the present study, it was not possible to examine the potential associations with variables pertaining to fathers stratified by child gender.

Participation in swimming was higher among children with mothers identifying as housewives or part-timers, while lower with junior high school or high school graduate mothers. Compared to full-time mothers, mothers who were full-time housewives or part-time workers might have more time to spare, enabling them to take their children to and from sports facilities, which may have resulted in a higher rate of participation in sports by their children. Although household income could not be obtained in the present study, it is possible that the junior high school or high school graduate mothers are related to the lower household income. A previous systematic review has indicated that SES and neighborhood residence could be associated with accessibility to swimming facilities and engagement in swimming lessons [16]. Furthermore, barriers to preschool children’s participation in swimming lessons in Australia included the affordability of swimming lessons and lack of or poor access to swimming lessons [17]. Therefore, unlike other sports, it may be challenging to participate in swimming as a leisurely activity, and parents need to deliberately create opportunities for their children to engage in swimming. In our sample, mothers who identified as housewives or part-timers may have been able to take their children to swimming lessons in distant facilities and have the flexibility to fit in lesson schedules. Swimming is likely to be organized through swimming clubs, as reflected in the ranking of activities offered by youth sports organizations and community sports clubs [18]. Based on the number of registered organizations for each sport type included in this study, the ranking from highest to lowest was softball, football, karate, track and field, baseball, swimming, gymnastics, and tennis. No organizations were found to be registered for dance or ballet [18]. According to the ranking of the number of comprehensive community sports clubs, ballet was not included in the ranking [19]. It was also reported that the average membership fee for a comprehensive community sports clubs was 695 yen (about 4.5 US dollars)/month [18]. On the other hand, membership fees for private swimming clubs were reported to be around ¥7000 (45 US dollars)/month [20]. Although paternal educational background was not associated with sports participation, the family’s economic situation may have an impact on participation in swimming.

Participation in football was higher among children with mothers employed part-time, but parental educational background was not, although a US study reported that parents with higher than a bachelor’s degree would be less likely to allow their children to participate in football (OR: 0.635) [21]. Hibshman and associates pointed out that master’s and doctorate-degree-holding parents are particularly risk-averse, while the present study was only able to examine university students and above.

Participation in gymnastics in our sample was higher among children with fathers who were junior high school or high school graduates. In Japan, gymnastics and rhythmic gymnastics have been reported to be events in which many infants and primary school children participate, and among sports injuries, unlike many other sports, injuries due to falls were high (24.4%), pointing to the influence of exercise style [22]. The discrepancy in results based on parental factors may, therefore, be due to perceptions of the risks associated with participating in gymnastics rather than economic reasons. On the other hand, examining the average annual income in the region revealed a tendency for lower average annual income to be linked to lower participation in football, while baseball participation was higher. Despite both being ball sports, the opposite trends observed are challenging to explain based on the information available from this study and previous research. For gymnastics, participation was notably higher only among children from regions with a middle-range average income.

For ballet, the participation rate was lower among mothers with a junior college or vocational school education compared to those with a university education or higher. The participation rate also tended to be lower among junior and senior high school graduates. In the present study, 96.6% of the participants were girls. In addition, no relationship was found between the mother’s educational background and dance in the present study. As mentioned above, there may be a route of succession from mother to daughter, especially for ballet among girls in this study [14]. Owen and associates [6] pointed out that sports participation involves costs such as registration fees, uniforms, transport, and equipment, which may be a major barrier for children from economically disadvantaged backgrounds. Compared to dance, ballet can be heavily influenced by SES, given the high lesson fees, the general price of equipment such as ballet shoes and leotards, and the cost of recitals.

This study has several limitations. First, generalizability is limited to the urban public primary schools that agreed to participate in this study. Second, due to the small number of fathers who participated, analyses involving fathers could not be carried out for girls. Third, although income is an important concept in discussing the relationship between inequality, class, and sports participation, it was not included in the survey items in this study. Fourth, this study is a cross-sectional survey conducted between 2012 and 2015, when the relative poverty rate for children in Japan was the highest. The results and arguments obtained are localized and require the accumulation of comparative studies in other places and with verification through longitudinal surveys.

This study showed differences in children’s sports participation rates by parents’ SES. The results provide some insight into measures that can be taken to provide more opportunities for children to participate in sports. For example, it may be useful to provide a supportive system for mothers with a full-time job.

## 5. Conclusions

The sports participation rate was higher among children with mothers identifying as housewives (All: OR = 2.91, Girls: OR = 1.82, Boys: OR = 5.69) or those with mothers employed part-time (All: OR = 2.90, Girls: OR = 1.76, Boys: OR = 5.69). Among girls, the continued sports participation rate was lower if their mothers were junior high school or high school graduates (OR = 0.25) or junior college/vocational school graduates (OR = 0.27). The odds of being a part of sports type like swimming were higher for children whose mothers had housewives or part-time jobs. Lower average community income was associated with lower participation in football and higher participation in baseball. These findings suggest that mothers’ employment and academic background are important correlates of sports participation for children, with variations observed by sport type and gender. Understanding the influence of SES on sports participation and the interaction between SES and gender can provide fundamental information for considering measures to reduce disparities and promote equitable access to organized sports participation in diverse populations.

## Figures and Tables

**Table 1:** Characteristics of participants.

		(*n*)	(%)	Sports Participation Rate (%)
Gender	Girls	269	51.3	66.9
	Boys	255	48.7	77.6
School grade	1st and 2nd grade	184	35.1	72.8
	3rd and 4th grade	203	38.7	73.4
	5th and 6th grade	137	26.1	69.3
Mother’s employment status	Unemployed	179	42.2	75.4
	Part-time	154	36.3	76.6
	Self-employed	23	5.4	52.2
	Full-time	68	16.0	52.9
Father’s employment status	Self-employed	38	14.4	71.1
	Full-time	226	85.6	72.6
Mother’s educational background	Junior high school and high school	82	18.6	65.8
	Junior college and vocational school	169	38.4	71.2
	University and above	189	43.0	73.3
Father’s educational background	Junior high school and high school	49	19.9	71.1
	Junior college and vocational school	30	12.2	73.3
	University and above	167	67.9	71.4
Regional annual income	Low group	163	31.1	68.7
	Middle group	202	38.5	74.8
	High group	159	30.3	72.3
Types	Swimming	196	37.4	
	Football	55	10.5	
	Gymnastics	41	7.8	
	Baseball	36	6.9	
	Dance	31	5.9	
	Ballet	29	5.5	
	Karate	19	3.6	
	Tennis	19	3.6	
	Athletics	15	2.9	

**Table 2:** The relationship between sports participation and socioeconomic status.

		All		Girls		Boys	
Sports Participants vs. Non-Sports Participants		OR	95% CI	OR	95% CI	OR	95% CI
Mother’s employment status	Unemployed	2.91	1.49–5.68 *	1.82	0.75–4.41 *	5.69	1.98–16.38 *
	Part-time	2.90	1.44–5.82 *	1.76	0.72–4.33 *	6.02	1.90–19.14 *
	Self-employed	1.25	0.42–3.77	0.34	0.07–1.75	5.00	0.83–30.00
	Full-time	reference		reference		reference	
Mother’s educational background	Junior high school and high school	0.59	0.30–1.15	0.44	0.19–1.03	0.72	0.23–2.27
	Junior college and vocational school	0.79	0.45–1.37	0.56	0.26–1.20	0.98	0.42–2.29
	University and above	reference		reference		reference	
Father’s employment status	Self-employed	0.88	0.29–2.62	0.92	0.14–5.99	0.85	0.21–3.33
	Full-time	reference		reference		reference	
Father’s educational background	Junior high school and high school	1.30	0.46–3.70	1.82	0.32–10.48	1.11	0.29–4.25
	Junior college and vocational school	0.57	0.18–1.83	0.79	0.15–4.10	0.47	0.09–2.54
	University and above	reference		reference		reference	
Regional annual income	Low group	0.85	0.52–1.38	0.81	0.42–1.54	0.90	0.43–1.89
	Middle group	1.14	0.71–1.85	1.16	0.62–2.19	1.12	0.54–2.31
	High group	reference		reference		reference	

Adjusting factors were sex, grade, and mother’s age in the analysis of mothers or father’s age in the analysis of fathers. OR: odds ratio, 95% CI: 95% confidence interval, *: *p*-value < 0.05.

**Table 3:** Relationship between participation in sports events and socioeconomic status.

Sports Participants vs. Non-Sports Participants			OR	95% CI
Swimming	Mother’s employment status	Unemployed	2.43	1.19–4.98 *
		Part-time	2.36	1.12–4.96 *
		Self-employed	0.78	0.22–2.85
		Full-time	reference	
	Mother’s educational background	Junior high school and high school	0.49	0.25–0.96 *
		Junior college and vocational school	0.93	0.57–1.53
		University and above	reference	
	Father’s employment status	Self-employed	0.50	0.15–1.69
		Full-time	reference	
	Father’s educational background	Junior high school and high school	0.80	0.29–2.21
		Junior college and vocational school	1.00	0.30–3.33
		University and above	reference	
	Regional annual income	Low group	0.98	0.62–1.55
		Middle group	0.99	0.64–1.54
		High group	reference	
Football	Mother’s employment status	Unemployed	3.61	0.76–17.18
		Part-time	5.19	1.05–25.70 *
		Self-employed	1.25	0.10–15.84
		Full-time	reference	
	Mother’s educational background	Junior high school and high school	0.79	0.25–2.49
		Junior college and vocational school	0.84	0.37–1.87
		University and above	reference	
	Father’s employment status	Self-employed	1.08	0.26–4.47
		Full-time	reference	
	Father’s educational background	Junior high school and high school	0.75	0.19–3.00
		Junior college and vocational school	0.42	0.05–3.85
		University and above	reference	
	Regional annual income	Low group	0.39	0.18–0.86 *
		Middle group	0.63	0.32–1.24
		High group	reference	
Gymnastics	Mother’s employment status	Unemployed	1.74	0.47–0.41
		Part-time	0.87	0.20–3.73
		Self-employed	2.82	0.49–0.25
		Full-time	reference	
	Mother’s educational background	Junior high school and high school	0.87	0.22–3.47
		Junior college and vocational school	2.22	0.91–0.08
		University and above	reference	
	Father’s employment status	Self-employed	2.88	0.58–14.24
		Full-time	reference	
	Father’s educational background	Junior high school and high school	5.16	1.03–25.88 *
		Junior college and vocational school	5.54	0.80–38.57
		University and above	reference	
	Regional annual income	Low group	1.05	0.37–2.99
		Middle group	2.96	1.24–7.06 *
		High group	reference	
Ballet	Mother’s employment status	Unemployed	1.45	0.38–5.45
		Part-time	0.78	0.18–3.35
		Self-employed	0.00	0.00–0.00
		Full-time	reference	
	Mother’s educational background	Junior high school and high school	0.29	0.07–1.14
		Junior college and vocational school	0.07	0.01–0.56 *
		University and above	reference	
	Regional annual income	Low group	1.27	0.48–3.36
		Middle group	0.90	0.34–2.41
		High group	reference	
Baseball	Regional annual income	Low group	2.77	0.99–7.76
		Middle group	3.02	1.12–8.14 *
		High group	reference	

Adjusting factors were sex, grade and mother’s age for analyses of mothers and father’s age for analyses of fathers, OR: odds ratio, 95% CI: 95% confidence interval, *: *p*-value < 0.05. Only mothers and regional annual income were analyzed for ballet and regional annual income for baseball.

## Data Availability

The raw data supporting the conclusions of this article will be made available by the authors upon request. The data are not publicly available due to ethical restrictions.

## Supplementary Materials

The following supporting information can be downloaded at https://www.mdpi.com/article/10.3390/sports13060165/s1, Table S1: The relationship between sports participation since preschool and socioeconomic status.

## Abbreviations

The following abbreviations are used in this manuscript:

SES	Socioeconomic Status
OR	Odds Ratio
