# Correction: Tanaka et al. Relationship Between Socioeconomic Status and Organized Sports Among Primary School Children: A Gender-Based Analysis of Sports Participation. *Sports* 2025, *13*, 165

**DOI:** 10.3390/sports14010002

**Published:** 2025-12-30

**Authors:** Chiaki Tanaka, Eun-Young Lee, Shigeho Tanaka

**Affiliations:** 1 Department of Human Nutrition, Tokyo Kasei Gakuin University, 22 Sanbancho, Chiyoda-ku, Tokyo 102-8341, Japan; 2 National Institute of Health and Nutrition, National Institutes of Biomedical Innovation, Health and Nutrition, Settsu, Osaka 566-0002, Japan; tanaka.shigeho@eiyo.ac.jp; 3 Institute of Nutrition Sciences, Kagawa Nutrition University, 3-9-21 Chiyoda, Sakado, Saitama 350-0288, Japan; 4 School of Kinesiology and Health Studies, Queen’s University, Kingston, ON K7L3N6, Canada; eunyoung.lee@queensu.ca; 5 Faculty of Nutrition, Kagawa Nutrition University, 3-9-21 Chiyoda, Sakado, Saitama 350-0288, Japan

## 1. Error in Table

In the original publication [1], there were values that were mistyped in Table 3 as published. For “Gymnastics”, the 95% CI for the variable of the “Mother’s employment status” being “Part-time” is “0.20–3.73”, not “0.20–0.85 *”. Similarly, the 95% CI for the variable of the “Mother’s educational background” being “Junior high school and high school” is “0.22–3.47”, not “0.22–0.84 *”. The corrected Table 3 appears below.

## 2. Text Correction

In accordance with the above corrections, there were errors in the original publication.

A correction has been made to Section 3, paragraph 4:

In terms of parental education, the odds of participation in swimming among children were lower with mothers reporting a junior high school or high school education (OR: 0.49), and ballet when mothers reported a junior college or vocational school education (OR: 0.07). On the other hand, the odds of participation in gymnastics were higher when fathers had a junior high school or high school education (OR: 5.16).

A correction has been made to Section 4, paragraph 8:

Participation in gymnastics in our sample was higher among children with fathers who were junior high school or high school graduates.

The authors state that the scientific conclusions are unaffected. This correction was approved by the Academic Editor. The original publication has also been updated.

## Figures and Tables

**Table 3:** Relationship between participation in sports events and socioeconomic status.

Sports Participants vs. Non-Sports Participants			OR	95% CI
Swimming	Mother’s employment status	Unemployed	2.43	1.19–4.98 *
		Part-time	2.36	1.12–4.96 *
		Self-employed	0.78	0.22–2.85
		Full-time	reference	
	Mother’s educational background	Junior high school and high school	0.49	0.25–0.96 *
		Junior college and vocational school	0.93	0.57–1.53
		University and above	reference	
	Father’s employment status	Self-employed	0.50	0.15–1.69
		Full-time	reference	
	Father’s educational background	Junior high school and high school	0.80	0.29–2.21
		Junior college and vocational school	1.00	0.30–3.33
		University and above	reference	
	Regional annual income	Low group	0.98	0.62–1.55
		Middle group	0.99	0.64–1.54
		High group	reference	
Football	Mother’s employment status	Unemployed	3.61	0.76–17.18
		Part-time	5.19	1.05–25.70 *
		Self-employed	1.25	0.10–15.84
		Full-time	reference	
	Mother’s educational background	Junior high school and high school	0.79	0.25–2.49
		Junior college and vocational school	0.84	0.37–1.87
		University and above	reference	
	Father’s employment status	Self-employed	1.08	0.26–4.47
		Full-time	reference	
	Father’s educational background	Junior high school and high school	0.75	0.19–3.00
		Junior college and vocational school	0.42	0.05–3.85
		University and above	reference	
	Regional annual income	Low group	0.39	0.18–0.86 *
		Middle group	0.63	0.32–1.24
		High group	reference	
Gymnastics	Mother’s employment status	Unemployed	1.74	0.47–0.41
		Part-time	0.87	0.20–3.73
		Self-employed	2.82	0.49–0.25
		Full-time	reference	
	Mother’s educational background	Junior high school and high school	0.87	0.22–3.47
		Junior college and vocational school	2.22	0.91–0.08
		University and above	reference	
	Father’s employment status	Self-employed	2.88	0.58–14.24
		Full-time	reference	
	Father’s educational background	Junior high school and high school	5.16	1.03–25.88 *
		Junior college and vocational school	5.54	0.80–38.57
		University and above	reference	
	Regional annual income	Low group	1.05	0.37–2.99
		Middle group	2.96	1.24–7.06 *
		High group	reference	
Ballet	Mother’s employment status	Unemployed	1.45	0.38–5.45
		Part-time	0.78	0.18–3.35
		Self-employed	0.00	0.00–0.00
		Full-time	reference	
	Mother’s educational background	Junior high school and high school	0.29	0.07–1.14
		Junior college and vocational school	0.07	0.01–0.56 *
		University and above	reference	
	Regional annual income	Low group	1.27	0.48–3.36
		Middle group	0.90	0.34–2.41
		High group	reference	
Baseball	Regional annual income	Low group	2.77	0.99–7.76
		Middle group	3.02	1.12–8.14 *
		High group	reference	

Adjusting factors were sex, grade and mother’s age for analyses of mothers and father’s age for analyses of fathers, OR: odds ratio, 95% CI: 95% confidence interval, *: *p*-value < 0.05. Only mothers and regional annual income were analyzed for ballet and regional annual income for baseball.
