# Supplementary material for: Relationship Between Socioeconomic Status and Organized Sports Among Primary School Children: A Gender-Based Analysis of Sports Participation
Source: Sports (Basel). 2025 May 28;13(6):165. doi: 10.3390/sports13060165 (PMC12196842; doi:10.3390/sports13060165)
Supplement: Supplementary file 1 [file sports-13-00165-s001.zip › sports-3572151-supplementary.pdf]

Supplementary material 1 The relationship between sports participation since preschool and socioeconomic status

		All				Girls					Boys			
Sports Participants vs. Non-Sports Participants		OR	95% CI			OR	95% CI				OR	95% CI		
Mother's employment status	Unemployed	0.93	0.32	-	2.69	1.28	0.28	-	5.93		0.92	0.19	-	4.57
	Part-time	1.09	0.37	-	3.19	0.61	0.15	-	2.58		1.98	0.36	-	10.94
	Self-employed	0.20	0.04	-	1.14	0.11	0.01	-	2.11		0.27	0.03	-	2.64
	Full-time	reference				reference					reference			
Mother's educational background	Junior high school and high school	0.50	0.20	-	1.24	0.25	0.06	-	0.99	*	1.00	0.27	-	3.73
	Junior college and vocational school	0.83	0.41	-	1.69	0.27	0.08	-	0.91	*	1.56	0.62	-	3.94
	University and above	reference				reference					reference			
Father's employment status	Self-employed	0.51	0.12	-	2.21	-	-	-	-	-	0.34	0.06	-	1.84
	Full-time	reference				-					reference			
Father's educational background	Junior high school and high school	1.13	0.28	-	4.51	-	-	-	-	-	1.86	0.40	-	8.76
	Junior college and vocational school	2.42	0.22	-	26.93	-	-	-	-	-	1.26	0.07	-	24.31
	University and above	reference				-					reference			
Regional annual income	Low group	1.38	0.74	-	2.59	1.55	0.55	-	4.38		1.30	0.59	-	2.90
	Middle group	1.18	0.65	-	2.12	0.95	0.39	-	2.35		1.38	0.64	-	3.01
	High group	reference				reference					reference			

Adjusting factors were sex, grade, and mother's age in the analysis of mothers or father's age in the analysis of fathers. B: regression coefficient, SE: standard error, OR: odds ratio, 95% CI: 95% confidence interval, *: p-value<0.05.
